# Carbon Nanotube and Cellulose Nanocrystal Hybrid Films

**DOI:** 10.3390/molecules24142662

**Published:** 2019-07-23

**Authors:** Mingzhe Jiang, Robert Seney, Paul Charles Bayliss, Christopher L. Kitchens

**Affiliations:** Department of Chemical and Biomolecular Engineering, Clemson University, Clemson, SC 29634, USA

**Keywords:** cellulose nanocrystals, carbon nanotubes, hybrid films

## Abstract

The use of cellulose nanocrystals (CNC) in high performance coatings is attractive for micro-scale structures or device fabrication due to the anisotropic geometry, however CNC are insulating materials. Carbon nanotubes (CNT) are also rod-shaped nanomaterials that display high mechanical strength and electrical conductivity. The hydrophobic regions of surface-modified CNC can interact with hydrophobic CNT and aid in association between the two anisotropic nanomaterials. The long-range electrostatic repulsion of CNC plays a role in forming a stable CNT and CNC mixture dispersion in water, which is integral to forming a uniform hybrid film. At concentrations favorable for film formation, the multiwalled nanotubes + CNC mixture dispersion shows cellular network formation, indicating local phase separation, while the single-walled nanotube + CNC mixture dispersion shows schlieren texture, indicating liquid crystal mixture formation. Conductive CNT + CNC hybrid films (5–20 μm thick) were cast on glass microscope slides with and without shear by blade coating. The CNT + CNC hybrid films electrical conductivity increased with increasing CNT loadings and some anisotropy was observed with the sheared hybrid films, although to a lesser extent than what was anticipated. Percolation models were applied to model the hybrid film conductivity and correlate with the hybrid film microstructure.

## 1. Introduction

With excellent mechanical, thermal and electrical properties, carbon nanotubes (CNT) have a plethora of potential applications [1,2]. The high aspect ratio of CNT offer wider opportunities for processing materials with applied shear to introduce anisotropic properties; a critical advantage that can be taken advantage of in colloidal nanomaterial dispersion processing [3,4]. Assembly of CNT and other anisotropic nanomaterials into ordered and oriented phases are highly desirable for many applications and are most easily achieved via colloidal processing [1], but polydispersity and poor colloidal dispersion in both aqueous and nonaqueous solvents make this a grand challenge. Methods to enhance CNT dispersion in aqueous solvents have relied on surface functionalization or modification of the dispersing medium. In the case of chemical modification, the structure and physical properties of CNT are altered. Lyotropic liquid crystal phase behavior of CNT and other nanoscale rigid rods has been observed in superacids [5], but the handling in superacids is hazardous and prohibitive for large scale applications. An alternative route to disperse CNT is adding a dispersant surfactant or polymer to generate noncovalent functionalization [6,7,8]. One example is the use of denatured DNA to promote a CNT lyotropic liquid crystal dispersion [9]. This work aims to investigate the use of benign, anisotropic colloidal nanorods for dispersion enhancement and the creation of conductive hybrid films.

Cellulose is the most abundant natural polymer in the biosphere. It is desirable for many material applications because it is an inexpensive and abundant biopolymer with certain useful characteristics including biodegradability, biocompatibility, nontoxicity, and mechanical strength [10]. Cellulose nanocrystals (CNC) are isolated from native cellulosic materials by controlled acid hydrolysis, where surface sulfate groups impart excellent colloidal stability and rich liquid crystal phase behavior [11]. The lyotropic phase behavior brings an anisotropic orientation, which is significant for a number of potential applications [12,13,14]. For example, the use of CNC in high performance coatings is attractive for micro-scale structures or device fabrication largely due to the anisotropic, or rod-shaped geometry [15].

CNC have been evaluated as dispersing agents for CNT where the CNC (200) surface plane displays hydrophobic character due to predominant C−H moieties [16,17]. These hydrophobic facets interact with hydrophobic CNT and aid in colloidal dispersion through hydrophobic association. The long-range electrostatic repulsion plays a substantial role in forming a stable CNT/CNC water dispersion. Since CNC do not completely cover the CNT surface, percolation can occur allowing for hybrid thin films to display electrical conductivity [18]. The hybrid assembly has potential to exploit the CNC anisotropy, allowing a combination of the attractive features of both CNC and CNT, i.e., anisotropic mechanical properties and electrical properties [19]. Taking full advantage of CNC to boost the physical properties of CNT composites can provide new design opportunities for next-generation devices with low-cost, lightweight, foldable, and portable electronics.

In this study, multiwall carbon nanotubes (MWNT) and single-wall carbon nanotubes (SWNT) were dispersed in water with CNC functionalized with sulfate half-esters as the dispersing agent. The stability and dispersing capacity of the CNC assisted MWNT and SWNT aqueous dispersions was characterized. The dispersion phase behavior at higher concentration exhibited a cellular network structure with intermittent liquid crystal phases for the MWNT/CNC mixture, but a liquid crystal mixture for SWNT/CNC mixture. The CNT and CNC hybrid film was cast using a blade coating technique and its microstructure was examined. The hybrid film conductivity was correlated with the incorporated CNT concentration, structure, and orientation. 

## 2. Results and Discussion

### 2.1. Dispersion of MWNT and SWNT with CNC in Water

**Figure 1:**
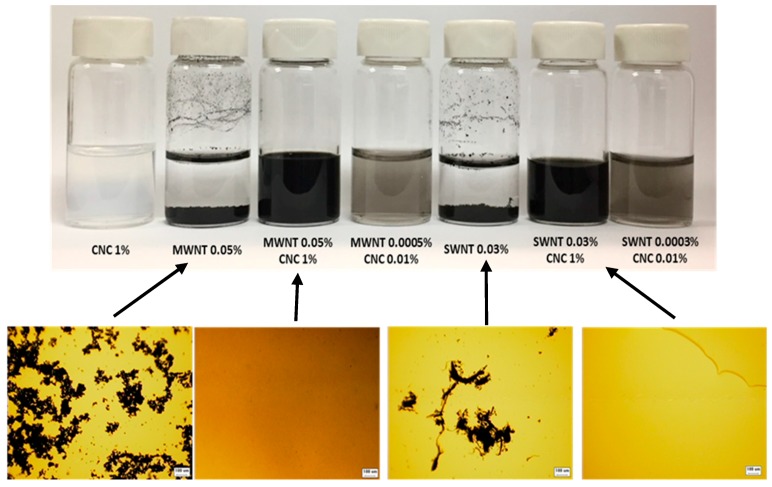
Vials (6 mL) containing aqueous dispersions of (left to right) cellulose nanocrystals (CNC) 1%, multiwall carbon nanotubes (MWNT) 0.05% at pH = 3.1, MWNT 0.05% and CNC 1%, MWNT 0.0005% and CNC 0.01%, single-wall carbon nanotubes (SWNT) 0.05% with pH = 3.1, SWNT 0.05% with CNC 1%, and SWNT 0.0005% with CNC 0.01%. All samples were imaged after sitting for 6 weeks. Optical microscope images of coordinating dispersions are shown below to demonstrate dispersion. Scale bar: 100 µm.

The dark, homogeneous CNT/CNC dispersions are shown in Figure 1. The initial dispersion of both MWNT/CNC (0.05%wt/1%wt) and SWNT/CNC (0.05%wt/1%wt) is stable on the order of weeks after removal of the initial aggregates and nondispersed CNT by centrifugation. No observed precipitation was observed in the dispersions after six weeks. Control experiments are carried out with MWNT (0.05%wt) or SWNT (0.05%wt) at pH = 3.1 (equal to the CNC 1%wt dispersion), which show large coagulated bundles that quickly settle from suspension. This phase behavior demonstrates that CNC play a critical role in the MWNT and SWNT dispersion stabilization. An optical microscopy image of the dispersion is shown in Figure 1. The MWNT or SWNT bundles or agglomerates can be easily seen in the samples without CNC, but with CNC at the same CNT concentration, the dispersions show no obvious bundles or agglomerates under 20× magnification. 

A series of parametric experiments were performed to optimize the CNT dispersion yields. The UV–Vis absorbance of suspensions after centrifugation was measured to determine the CNT dispersion. Analysis of various concentrations of CNC/MWNT or CNC/SWNT demonstrated that CNC effectively dispersed both MWNT and SWNT in aqueous media. 

**Figure 2:**
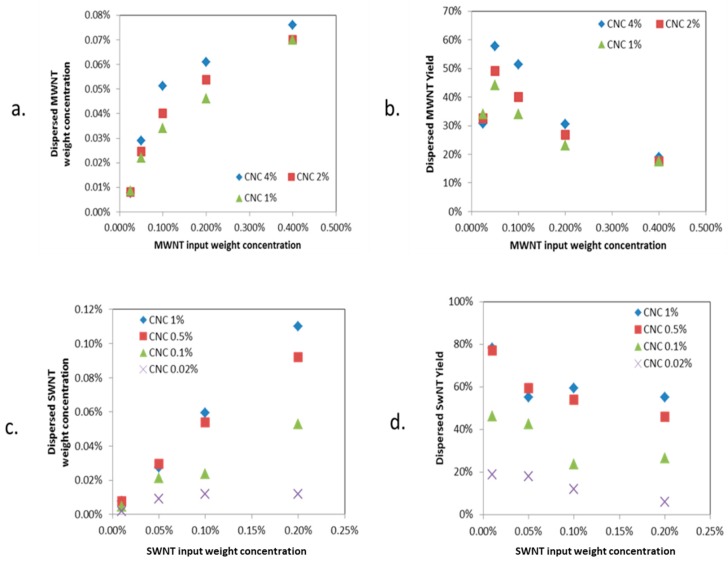
MWNT (**a**,**b**) and SWNT (**c**,**d**) dispersed concentration and yield vs. initial added CNT concentration with different CNC concentrations.

**Figure 3:**
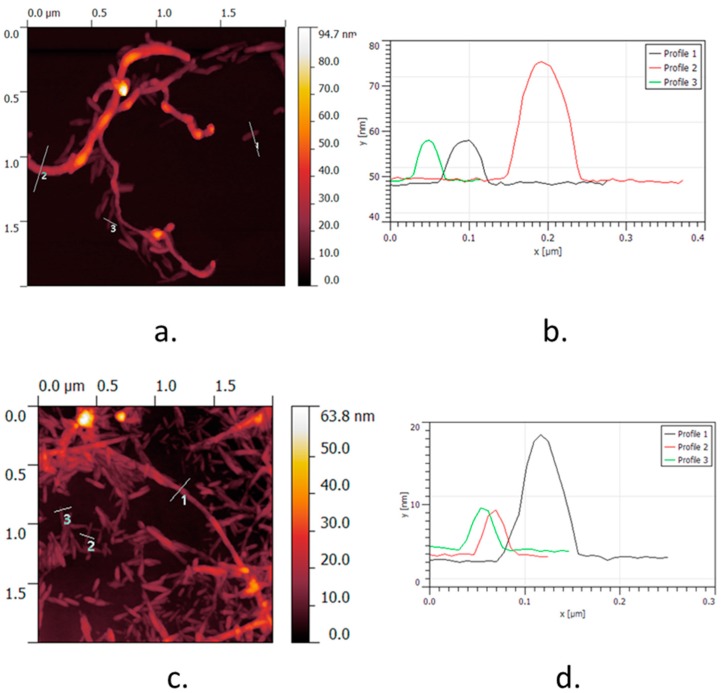
(**a**,**b**) Atomic force microscope height image of the MWNT/CNC complex and its associated height profiles. (**c**,**d**) Atomic force microscope height image of the SWNT/CNC complex and its associated height profiles.

For MWNTs (Figure 2a,b), the concentrations included 1%wt CNC, 2%wt and 4%wt CNC each with 0.025%wt, 0.05%wt, 0.1%wt, 0.2%wt, and 0.4%wt MWNT in water. Figure 2 shows the dispersion concentration and CNT yield after the final sonication where the dispersed CNT concentration increases with increasing CNT initial concentration at all CNC concentrations. Figure 2a,b shows a maximum MWNT dispersion yield at the initial MWNT concentration of 0.05%wt. Above 0.05%wt, the MWNT dispersion yield decreases with increasing MWNT initial concentration for all CNC concentrations. This critical MWNT concentration for maximum yield was also observed by Richa et al. [20], with MWNT dispersed in water with other surfactants (Triton X-100, Tween 20, and sodium dodecyl sulfate (SDS)), all showing maximum yield at low MWNT initial concentrations. In Figure 2c,d, the initial CNC concentration was varied from 0.02 to 4%wt for SWNT initial concentrations from 0.01 to 0.2%wt. As with MWNT, the dispersed SWNT concentration increases with increasing SWNT initial concentration at all CNC concentrations. SWNT dispersion yield was greatest at the lowest SWNT initial concentration studied, 0.01%. The SWNT dispersion yield decreased with increasing SWNT initial concentration at all CNC concentrations. Both SWNT dispersion concentration and yield increase as CNC concentration increases at fixed SWNT initial concentration. The maximum dispersing SWNTs yield is 80% (Figure 3c). A yield of 50% is reported by Haggenmueller et al. [21] with sodium dodecyl benzene sulfonate (SDBS) and SDS under similar conditions (sonication time: 2 h, 1.5 W/mL; centrifugation: 21,000× *g* for 2 h; initial SWNT concentration: 0.5 g/L with a ratio of SWNTs/surfactant by mass of 1:10). A higher yield of 88% was reported by Olga et al. [22] with sodium dodecylbenzenesulfonate (NaDDBS) at a lower SWNT dispersion concentration (0.006%wt). The CNT dispersing behavior is presumably due to the size polydispersity and surfactant to surface area ratio. The dispersion yield is a function of the CNT dispersibility and surfactant or CNC noncovalent association. For a stable dispersion, a concentration is attained for which the surfactant or CNC concentration is sufficient to disperse the CNT; i.e., the maximum yield limit is achieved. For subsequent increases in surfactant or CNC, the concentration of dispersed nanotubes will increase but not proportionately, leading to a decreased yield. These results are consistent with Jean-Bruno et al. [23], who proposed that the hydrophobic phase of CNC interacts with the carbon nanotube and aids in dispersion in water. The higher CNC concentration should lead to a proportionate increase in CNT dispersion, however the decreasing trend of the CNT dispersion yield indicates there are other parameters at play, which are less dependent on the CNC concentration and more so on available free volume. 

To understand the dispersion mechanism, AFM images of MWNT/CNC and SWNT/CNC from dilute dispersion are shown in Figure 3. It is reported that the alignment of CNC along the length of SWNT is attributed to hydrophobic interactions that take place between the (200) surface crystalline plane of the amphiphilic CNC and SWNT sidewall. In the case of MWNT/CNC hybrids, AFM observations show that CNC are not necessarily aligned along the MWNT sidewall, but rather tangential. The MWNTs have a diameter of 5 to 10 nm, so their surface curvature is much less than that of SWNTs (1–2 nm). Therefore, the hydrophobic interactions between MWNTs and CNC do not require a perfect alignment of both objects. Moreover, the MWNTs have many defects and increased flexibility, resulting in more curvature over the length (micrographs not shown here). These defects are associated with oxidation, which leads to the presence of hydrophilic groups on the sidewall, decreasing the effective hydrophobic interaction strength. Elongated structures of several hundred nanometers can be attributed to CNT, and those with a length of ~120 nm can be attributed to CNC. This is confirmed by the height profiles recorded across the CNT, as shown in Figure 3b. 

### 2.2. Phase Transition of Concentrated CNC/MWNT and CNC/SWNT Dispersion in Water

**Figure 4:**
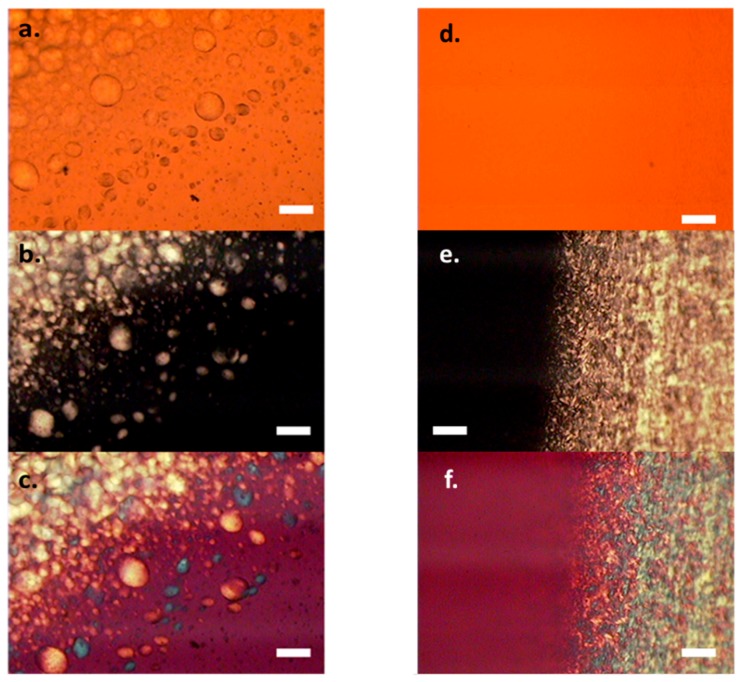
Optical microscope images of 8%wt. CNC with 0.16%wt. MWNT dispersion (**a**–**c**) and 8%wt. CNC with 0.16%wt. SWNT dispersion (**d**–**f**). The images include normal light (**a**,**d**), polarized light (**b**,**e**) and polarized light with first-order retardation plate (**c**,**f**). Scale bar: 50 μm.

Aqueous dispersions of sulfonated CNC exhibit lyotropic phase behavior, readily transitioning from isotropic to a biphasic suspension and finally to a liquid crystalline phase with increasing concentration [24]. It has been reported that dispersing CNT in a liquid crystal will show a new mixture of liquid crystal dispersion [25,26]. Thus, the CNT/CNC dispersion has potential to exhibit intrinsic liquid crystal like structure at concentrated regions. Usually, the phase behavior of lyotropic or mixture systems is studied in cuvettes and observed between cross-polarized light to detect the birefringence. However, due to the dark color of the CNT, the birefringence texture is been blocked at normal cuvette with thickness of 2 mm. To overcome this, suspension droplets were prepared with conventional microscope slide and cover glass with 120 μm spacer (very short light pathlength) and observed by cross-polarized microscopy. Ordered regions did appear as the water evaporated from the edges of the solidifying sample. Representative cross-polarized optical microscopy images of the ordered regions close to the edge of both MWNT/CNC and SWNT/CNC dispersions are shown in Figure 4 to represent the mixture dispersion phase transition at relatively high concentrations. 

For MWNT/CNC dispersion at a 1:50 volume mixing ratio and overall concentration of 8.0%wt, birefringent droplets were observed within a continuous isotropic phase (viewed as black between crossed polars). The birefringence within the droplets indicates the formation of liquid crystal polydomain (known as tactoid, spherical shape assembly with chiral nematic order) [27], which is consistent with pure CNC suspensions at the same concentration [24]. First-order retardation plate cross-polarized microscope image in Figure 4c further supports this birefringent texture, showing pure blue or yellow color inside each cell, indicating orientation order. 

The transparency inside the droplet and the dark color at the droplet interface under normal light in Figure 4a indicates the accumulations of MWNT at the interface, suggesting phase separation between CNC and MWNT at higher concentrations. This phase behavior has been observed at multiple mixing ratios of MWNT/CNC dispersions. No other phase state has been found in the MWNT/CNC dispersion at high concentration. 

For SWNT/CNC dispersion at a 1:50 mixing ratio and concentration of 8.0%wt, under normal light there are no detectable SWNT bundled agglomerations (Figure 4d). Unlike MWNT/CNC, the SWNT/CNC dispersion maintains a stable dispersion at high concentration. In polarized microscope images, the presence of droplets and dark interface are absent. Instead, schlieren textures are observed, which are indicative of a nematic phase. The full-wave plate cross-polarized microscope image in Figure 4f supports this nematic texture with blue and yellow color representing local particle alignment. It need be noted that this schlieren texture (nematic orientation) is observed at relatively low SWNT concentrations, since the high loading SWNT dispersions (SWNT loading > 6%) are not light transparent. The uniform liquid crystal formation with SWNT/CNC dispersions can be attributed to the aligned attachment of CNC along the length of SWNT as discussed in last section. This is consistent with the concept of liquid crystals assisting CNT orientation reported in other literature [28]. On the other hand, for MWNT, CNC have a tangential attachment, which may not aid in aligning the MWNT long axes in the same direction of CNC orientation director. More evidence of the microstructure for both MWNT/CNC and SWNT/CNC hybrid are discussed for the dried film characterization.

### 2.3. Properties of MWNT/CNC and CNC/SWNT Hybrid Films

**Figure 5:**
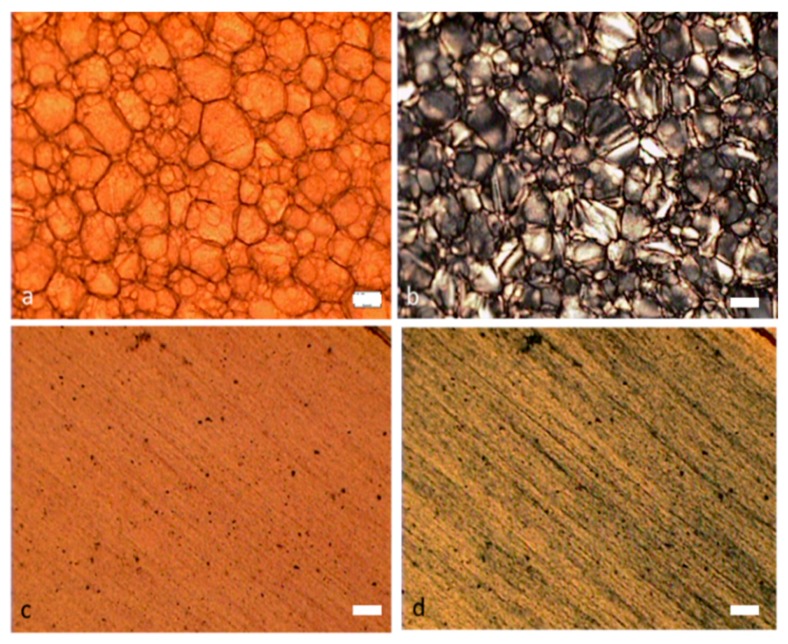
Optical microscope images of MWNT/CNC (2% MWNT and 98% CNC) hybrid films. (**a**,**c**) are normal light optical microscope images of (**a**) sedated dried CNC/MWNT hybrid film and (**c**) blade-coated dried film; (**b**,**d**) are polarized light optical microscope images of (**b**) sedated dried CNC/MWNT hybrid film and (**d**) blade-coated dried film. scale bar: 20 µm.

**Figure 6:**
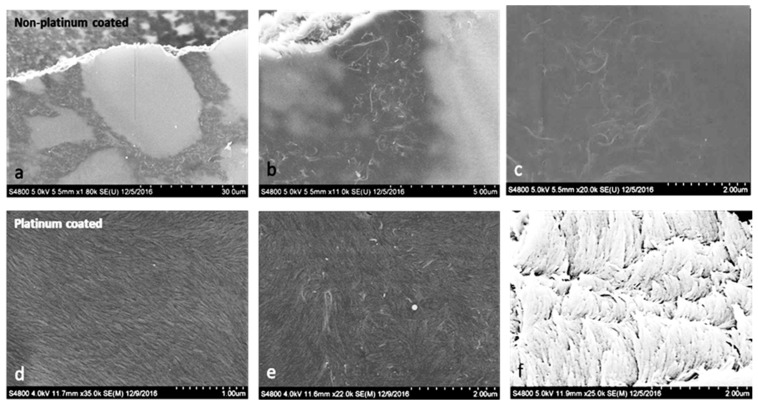

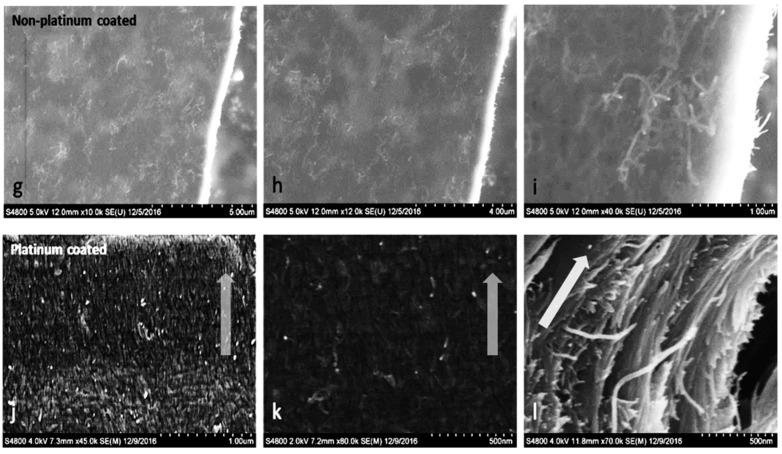
SEM images of 2% MWNT and 98% CNC hybrid films. (**a**–**c**) Images of a droplet dried hybrid film without platinum coating. (**d**–**f**) Images of a sedated dried hybrid film with platinum coating. (**g**–**i**) Blade-coated film without platinum coating. (**j**–**l**) Images of images of a blade-coated film with platinum coating. The arrow represents the blade shear direction. (The image contrast has been adjusted from the original image to better show the structure formation.)

Figure 5 shows optical microscopy images on the surface of MWNT/CNC sedated dried films (a,b) and blade sheared dried films (c,d). For MWNT/CNC sedated dried film, a cellular structure was formed with regions of CNC liquid crystals bounded by MWNTs at interfaces. Figure 6b shows the finger print birefringence texture is not observed from the MWNT/CNC sedated dried film under polarized light, indicating that the MWNT/CNC remain at the intermediate stage of tactoid formations and never evolves into long range chiral nematic order. It could be because that the MWNT covered on the interface seals each tactoid domain and prevent it from being further interconnected to each other to form chiral nematic network. Additionally, this cellular network has been observed in different loading concentration in the entire measured range (0.5 to 15%). Figure 6 provides SEM imaging of the film surface with and without metallization. SEM imaging with and without metallization is an excellent tool to distinguish conducting fillers in an insulating matrix. Because the CNC are insulators, metallization is mandatory to avoid charging effects and obtain well-resolved SEM micrographs. In Figure 6a–c without metallization, the CNC are not resolved but a loose network of long, bright filaments are observed, identifying the conductive nanotubes. Figure 6a clearly shows the aggregated MWNTs forming the outline of each cellular structure, which constitutes a percolated network on the film surface. This percolation network is contributing to the electrical conductivity of film, that will discuss in later of the section. Inside the cells, MWNTs are not observed, confirming phase separation between MWNT and CNC observed with polarized microscopy. Figure 6d–f, obtained after metallization, clearly shows the resolved CNC and the MWNTs to a lesser extent due to the low 2% MWNT loading fraction. It also shows orientation order of CNC and a plus/minus disclination pair, which confirmed the CNC liquid crystal phase formation. No long range left-handed helical structure [27] is observed, which may result from MWNT aggregation into the cellular structure that prevents liquid crystal network formation. The MWNT/CNC blade-sheared dried film clearly does not possess the cellular structure (Figure 5c,d), but rather a striped texture is observed, indicating that the liquid crystalline domains are elongated in the blade shear direction. SEM imaging (Figure 6g–i) without metallization shows that the aggregated MWNT cellular structure is disrupted into small disconnected random shape (isotropic) entangled MWNT aggregations. Figure 6j shows that the blade shear does aligning CNC (as the CNC oriented along the blade shear direction), but MWNT alignment is not observed.

**Figure 7:**
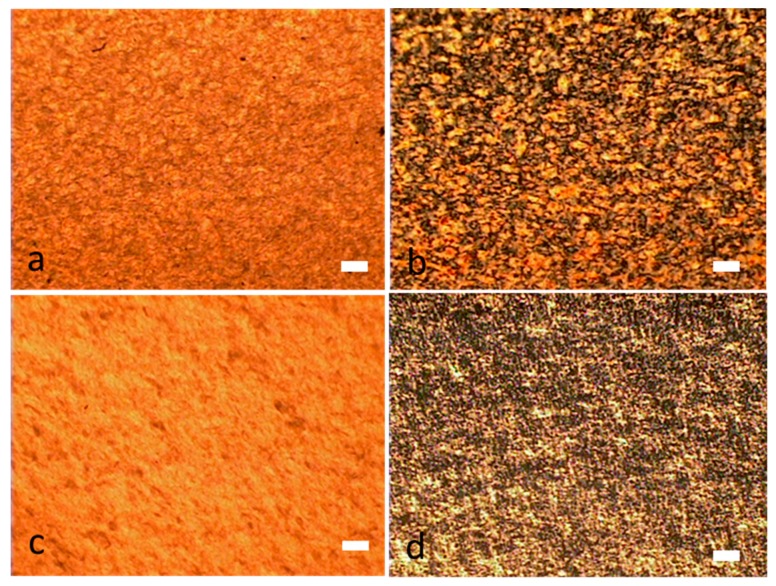
Optical microscope images of 2% SWNT and 98% CNC hybrid films. (**a**,**c**) Normal light optical microscope images of (**a**) sedated dried and (**c**) blade-coated hybrid films. (**b**,**d**) Polarized light optical microscope images of (**b**) sedated dried and (**d**) blade-coated hybrid films. Scale bar: 20 µm.

**Figure 8:**
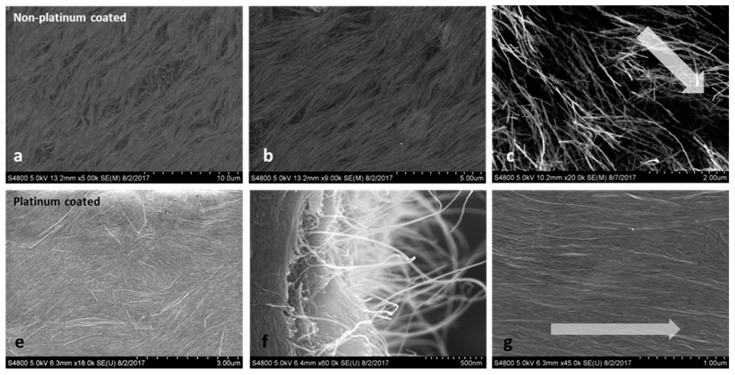
SEM images of 2% SWNT and 98% CNC hybrid films. (**a**,**b**) Images of sedated dried hybrid films without platinum coating. (**e**,**f**) Images of sedated dried hybrid film with platinum coated. (**c**,**g**) Images of a blade-coated film (**c**) without platinum coating and (**g**) with platinum coating. The arrow represents the blade shear direction.

Figure 7 shows optical microscope images of the surface of a 2% SWNT/CNC sedated dried film (a,b) and blade sheared dried film (c,d). Figure 8 shows the SEM image of the film surface, again obtained with and without metallization. For the SWNT/CNC sedated dried film, Figure 7b shows the schlieren texture under polarized light (similar to the texture observed in DNA stabilized carbon nanotube aqueous dispersion [9] and acid treated CNT liquid crystal structure [5]). This nematic orientation is confirmed in Figure 8a,b, which clearly shows SWNT orientation order for the sedated and sheared dried films. The SWNT and CNC are constrained to lie along the film surface plane direction so that disclinations have a wedge character and only the bend and the splay distortions are apparent. SEM imaging in Figure 8b shows the dried film free of disclinations in a local scale of 10 μm, which is direct evidence of highly ordered nematic orientation. Figure 8f also shows SWNT pull-outs as well as SWNT-reinforced CNC protruding layers, which is indicative of uniform dispersion within the dried film. For the blade sheared dried film, the schlieren texture and liquid crystal ordered structure has been disrupted, but the CNC alignment along the blade shear direction is enhanced but with discontinuous orientation domain, indicated by the birefringence texture observed in Figure 7d. Furthermore, SWNT alignment along the blade shear direction is not obvious as shown in Figure 7c. The ordered packing of SWNT is disrupted with blade shear application, instead of being enhanced in one direction. More results on the anisotropicity value of conductive percolation curve of the blade-coated CNC/SWNT film will be discussed in later section. 

### 2.4. Percolation of MWNT/CNC and CNC/SWNT Hybrid Films

According to statistical percolation theory, the dependence of the hybrid film conductivity, σ, on the conductive filler volume fraction, φ, can be described by a scaling law of the form

(1) $$\sigma =\sigma_{0}{\left(\varphi -\varphi_{c}\right)}^{t}$$

where $\sigma_{0}$ and $\varphi_{c}$ are the conductivity of the fillers and the percolation threshold, respectively [29]. In this theory, a sharp increase in conductivity is observed with increasing volume fraction of conductive fillers at the percolation threshold, which indicates the formation of a conductive pathways. The percolation threshold $\varphi_{c}$ is obtained by fitting Φc in a linear plot of experimental log σ versus log (Φ − Φc). The fitted critical exponent t is expected to depend on the system dimensionality with calculated values of t = 1.33 for two dimensions and t = 2 in three dimensions [30,31].

**Figure 9:**
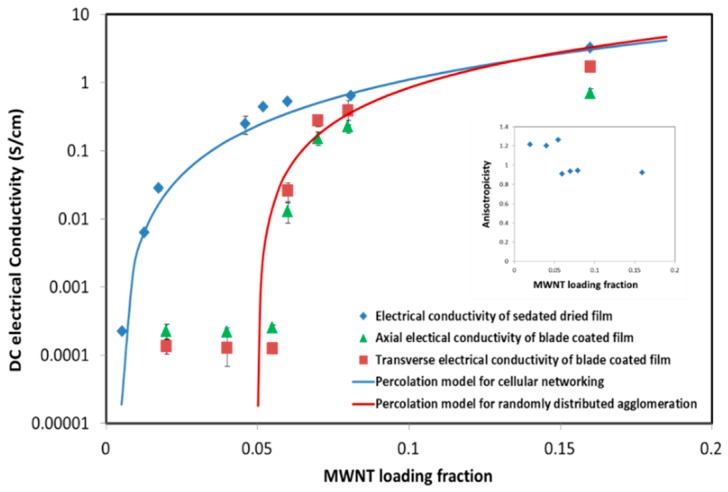
Electrical conductivity of sedated and blade sheared dried MWNT/CNC films as a function of MWNT volume fraction. The blade sheared film conductivity was measured in the direction of shear (axial) and perpendicular to shear (transverse). The inset is the anisotropicity (ratio of axial and transverse electrical conductivity) of blade-coated film conductivity at different MWNT loading fractions.

The MWNT/CNC hybrid film electrical conductivity were measured after conditioning at 24.9 °C and 45% relative humidity, as the conductivity has been shown to be a function of relative humidity [32]. The percolation behavior of both blade sheared and sedated dried films are shown in Figure 9. According to the optical microscope and SEM imaging, the MWNT cellular network is formed in sedated dried film. This cellular network is also found in SWNT/polystyrene composite films [33] and metal–insulator particle composites [34]. A cellular network model has been developed to fit the percolation, which can also be used here to explain MWNT/CNC sedated dried films. For the cellular structure in the MWNT/CNC sedated dried films, the model [35,36] describing the segregated conductive filler–matrix composites can be applied:

(2) $$\varphi_{c}=\frac{3W}{L\lambda }$$

where λ = D/d, D is the CNC micro domain size, d is the average MWNT aggregation size, and W/L is the MWNT aspect ratio. The effective nanotube size, d, is estimated as an average distance between the linkages of crossing nanotubes. From Figure 7, the average CNC domain size, D, is approximately 30 µm and d is approximated at 3 µm, yielding a D/d of approximately 10. The theoretical percolation threshold according to Equation (2) is 0.001–0.006 (for an aspect ratio range of 0.0025 to 0.02), which is consistent with the best fit experimental value of 0.0045 from the blue curve in Figure 9. The fitted critical exponent value of 2.09 suggests a three dimensional percolation system.

For the disrupted cellular network in the blade sheared MWNT/CNC film, an interparticle distance (IPD) model (Equation (3)) for an aggregated composite system [37] was applied for percolation threshold prediction.

(3) $$\varphi_{c}=\frac{\xi \varepsilon \pi }{6}+\frac{\left(1-\xi \right)27{\pi W}^{2}}{4L^{2}}$$

where ε is the localized volume fraction of MWNTs in an agglomerate and ξ is the agglomerated MWNT volume fraction. For blade sheared MWNT/CNC films, it is reasonable to set ξ to 1, since only agglomerated MWNTs are observed in the SEM image. According to the measurement of MWNT area percentage in SEM images yields ε equal to 0.1 and a theoretical percolation threshold of Φc is 0.051 from Equation (3). This value is similar to the experimental data fitting result of $\varphi_{c}=0.050$ with the fitting critical exponent t as 1.73 for the red curve in Figure 9. 

The axial percolation threshold in blade sheared film is 5.4%, which is one magnitude order higher than the sedated dried film. This is due to the agglomerated filler creating percolation pathways where the fillers intersect each other with multisite contact in misoriented aggregations. Moreover, the localized dense agglomerations of filler will inhibit the formation of spanning network of percolation in the whole composite system. Thus, a filler effective occupied volume, defined as the volume of the composite where the filler is concentrated. The volume that contains no filler does not contribute to the conductivity and percolation network formation. Thus, the lower the percentage of the effective occupied volume to the whole composite, the easier percolation network can be reached. Figure 8 shows cell-like network structure (indicating a phase separation between CNC and MWNT), which creates pathways for conducting electricity. Unlike the normal assumption of static percolation theory for well-dispersed conductive fillers, the MWNT aggregate phase separation decreases the effective MWNT-occupied volume in the composite film and thus an order of magnitude lower percolation concentration threshold. 

The effective MWNT-occupied volume in blade-coated composite film is the total volume of the film due to the disruption of two phases by shear force. The conductivity of the blade-coated film was measured in the axial and transverse directions to determine anisotropicity; the axial electrical conductivity to transverse conductivity ratio. When the MWNT concentration is below the percolation threshold, the axial conductivity is slightly higher than the transverse value, with anisotropicity below 2. This could also be correlated with sheathing effects (also named as tunneling effect). The aligned CNC generate more charge barriers between MWNTs, creating fewer electrical pathways formed in the transverse direction. Interestingly, above the MWNT percolation threshold, an average anisotropicity of 0.9 is observed, indicating greater conductivity in the transverse direction, which may be attributed to the observed tangential attachment of the CNC to the MWNT and the CNC orientation in the shear direction. 

**Figure 10:**
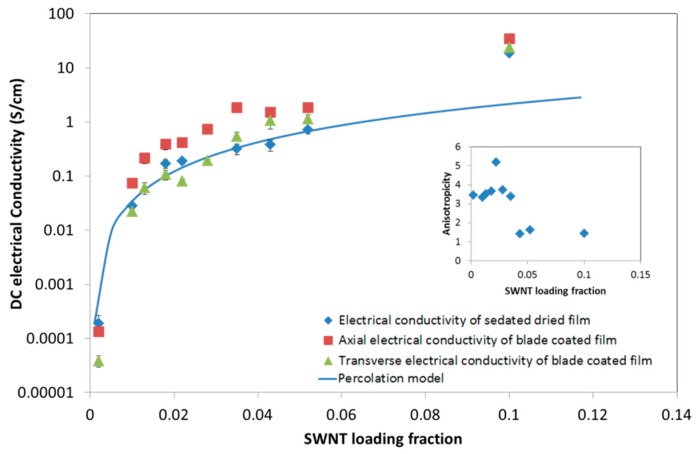
Electrical conductivity of sedated and blade sheared dried SWNT/CNC films as a function of SWNT volume fraction. The blade sheared film conductivity was measured in the direction of shear (axial) and perpendicular to shear (transverse). The inset is the anisotropicity (ratio of axial and transverse electrical conductivity) of blade-coated film conductivity at different SWNT loading fractions.

The SWNT/CNC hybrid film electrical conductivity was measured at 24.9 °C and 45% relative humidity and the percolation behavior of both sedated and blade-coated dried films are shown in Figure 10. Unlike the MWNT/CNC hybrid film, the percolation thresholds of the blade-coated and sedated dried film are consistent. This is attributed to the lack of observed phase separation in the SWNT/CNC film (below 10% SWNT loading). A statistical (well dispersed) randomly oriented cylinder model [38] is applied to the sedated dried film percolation prediction, with $\varphi_{c}=0.7W/L=0.0007$. This is consistent with the experimental fitting $\varphi_{c}=0.0005$ and critical exponent of 2.06, which fits with a correlation factor of 0.98 below 6% SWNT loading; below the upper percolation threshold range of approximately 0.05 to 0.1% in CNT composites in literature [29]. The critical exponent indicates a three dimensional percolation system. At 10% SWNT loading, the conductivity is an order of magnitude greater than the model prediction, which may be attributed to SWNT network formation of phase separation, negating the statistical percolation ‘well-dispersed’ assumption. Instead, SWNT entanglement and network formation generates additional conductive pathways. This phenomenon has been also found in carbon nanotube epoxy composites [39]. SEM images of higher SWNT loading film in Figure S6 in supporting information supports network formation.

The conductivity anisotropicity for the blade sheared films in Figure 10, did not effectively improve the electrical conductivity compared to sedated dried film, which indicates consistent homogeneous dispersion and a low level of SWNT orientation. The electrical anisotropicity is slightly higher than MWNT/CNC blade-coated film, with a maximum value of 5 at 0.02 SWNT fraction. Above 0.03 the anisotropicity approaches 1, which indicates no electrical property difference between the two directions. This agrees with the SEM imaging showing a lack of SWNT alignment in the shear direction. Thus, blade shear is not sufficient for SWNT alignment during the composite film processing. Applying magnetic or electric field for CNT alignment might be alternative way for anisotropicity improvement [40,41].

## 3. Materials and Methods

### 3.1. Preparation of Aqueous MWNT/CNC and SWNT/CNC Mixture Dispersion

Aqueous CNC suspensions were prepared by controlled sulfuric acid hydrolysis of cellulose filter paper by previously published methods [24]. The CNC particle density is 1.6 g/cm^3^. MWNTs (Stock#: 1236YJS; Density: 2.1 g/cm^3^; Purity: > 95%wt; outside diameter: 10–20 nm; inside diameter: 5–10 nm; length: 0.5–2 μm) and SWNT (Stock#: 1246YJS; Density: 1.6 g/cm^3^; Purity: > 90%wt; Outside Diameter: 1–2 nm; Inside Diameter: 0.8–1.6 nm; Length: 1–3 μm) were purchased from Nanostructure & Amorphous Material Inc. Both CNT were chosen with short lengths in order to prevent the entanglement of CNT during mixing and promote ordered orientation formation with the assistance of CNC and shear.

All dispersions were prepared by mixing desired concentrations of CNC and CNT in deionized water with a horn sonicator (a Fisher Scientific Model 550 Sonic Dismembrator) in an ice water bath to prevent temperature increase. For the MWNT study, CNC concentrations included 1.0, 2.0, and 4.0wt.% and MWNT concentrations included 0.025, 0.05, 0.1, 0.2, and 0.4wt.%. For the SWNT study, CNC concentrations included 0.02, 0.1, 0.5, and 1.0wt.% and SWNT concentrations included 0.01, 0.05, 0.1, and 0.2wt.%. The output power was fixed at 3 W/mL, and sonication time set to 40 min. The mixtures were then centrifuged at 15,600× *g* for 45 min. in a Thermo Scientific Sorvall Legend XTR Centrifuge to remove any MWNT or SWNT bundles and aggregates. The resulting aqueous MWNT/CNC and SWNT/CNC mixture concentration was adjusted by evaporating (under ambient condition (25 °C) with continuous stirring) or adding water. The concentration of the CNC or CNT/CNC dispersion was measured with a TA Instruments SDT Q 600 Thermogravimetric Analysis (TGA) and UV-Vis absorbance spectroscopy. In short, 50 µL of dispersion was loaded into a sample cup and heated to 110 °C at a rate of 20 °C/min and held isothermally for 20 min. The sample was then heated to 800 °C at a rate of 5 °C/min and then held isothermally for 10 min. This measurement was repeated in triplicate and averaged. To determine the CNC to CNT ratio, the same procedure was conducted but for the solids obtained from drying 0.5 mL of dispersion. 

Bundled carbon nanotubes do not contribute to the UV-Vis absorbance due to a lack of dispersion [42], enabling quantification of the dispersed CNT using UV-Vis absorption spectroscopy. A Varian Cary 50 Series UV-Vis spectrometer was used to quantify CNT dispersion by monitoring absorbance values at 710 nm. The linear Beer–Lambert law is well obeyed by MWNT and SWNT, as shown in Figures S1 and S2 in the supporting information. Samples were diluted 200-fold with water, resulting in certain MWNT or SWNT contents that were suitable for UV-Vis measurements. The blank used was the original CNC solution diluted by the same factor, under the same conditions as the samples themselves. The concentration was calculated according to the method developed by Haggenmueller et al. [21]. 

A Veeco Dimension 3100 atomic force microscope (AFM) equipped with a Nanoscope 3A controller in tapping mode in ambient air was used to analyze the morphology of MWNT/CNC or SWNT/CNC hybrids. A droplet of dilute dispersions (0.00003%wt MWNT/0.001%wt CNC/) and (0.00003%wt SWNT/0.001%wt CNC) was dried on a freshly cleaned silicon wafer substrate. Nanoscope III 5.12r3 software was used to image the CNC. The AFM tip (HQ:NSC15/AL BS) purchased from MikroMasch had a nominal radius of 8 nm, typical force constant of 40 N/m and typical resonance frequency of 325 kHz. MWNT/CNC or SWNT/CNC dispersions were characterized by optical microscopy using Olympus BX-60 optical polarized light microscope with cross-polarizers and full-wave plate. Samples were prepared with a conventional microscope slide and cover glass with a 120 μm spacer (CoverWell™ Perfusion Chambers) and sealed by fast-dry fingernail polish. Pictures were taken at 5 to 20× magnification between cross-polarizers at room temperature. 

### 3.2. Hybrid Film Casting and Characterization

The blade coating protocol began by pretreating glass microscope slides with piranha acid for 60 min. The treated slides were cleaned and then placed in an ultrasonic DI water bath for 20 min. To make the film, the microscope slide is preheated by a hot plate with a temperature of approximately 80 °C and the height-adjustable Teflon Coated Micron II Film Applicator was used to apply 0.3 mL of MWNT/CNC or SWNT/CNC mixture to cast the wetting film. A 10%wt CNT/CNC dispersion provided the desired viscosity needed to cast the films. The blade coater was immediately pulled across the microscope slide at a shear rate of ~500 s^−1^, with a height gap of 50 µm. The platform and slide then stayed on the hotplate (80 °C for 15 min), where the slide was allowed to dry. The sedated film is made by placing 2 mL of 1%wt SWNT/CNC or MWNT/CNC dispersion evenly on the cleaned slide surface and naturally drying at 20 °C without applied any external force. The naturally dried sedated films were dried in 20 °C for overnight and then put onto hotplate (80 °C for 15 min) to remove residue water. Electrical conductivities of hybrid films were measured after conditioning in a room controlled at 24.9 °C and 75% relative humidity with a Keiley 616 Digital Electrometer using a two-probe method, with silver paint in conjunction between the two ends of the hybrid film and copper wires for electrical contacts. Films with each loading fraction were prepared with three replicas and the mean and standard deviation are reported.

MWNT/CNC or SWNT/CNC films were characterized by optical microscopy using Olympus BX-60 optical polarized light microscope with cross-polarizers and full-wave plate. Scanning electron microscopy (SEM) images of dried hybrid films with magnification up to 300 K were acquired on a Hitachi S-4800 Type II Ultra-High Resolution Field Emission SEM operating at 2 keV and working distance 5.7 mm. Thin (~2 nm) Pt layer coated and noncoated films were both examined by SEM to detect the insulated CNC and conductive SWNT or MWNT.

## 4. Conclusions

This study investigates the dispersion of CNT in aqueous CNC-SA suspensions and the ability to cast hybrid CNC films with electrical conductivity. The hydrophobic regions of surface-modified CNC can interact with hydrophobic CNT, and the CNC electrostatic repulsion aids in forming a stable CNT/CNC water dispersion. MWNT/CNC aqueous suspension reaches its maximum dispersion yield of 60%, while SWNT/CNC aqueous suspension reaches its maximum dispersion yield of 80%. In the measured loading concentration range (<15%), the MWNT/CNC dispersion shows cellular network formation, indicating mixture phase separation. In contrast, SWNT/CNC dispersions show schlieren texture, indicating a liquid crystal mixture formation and a stable homogeneous dispersion. These have been confirmed by SEM imaging of sedated dry films, in which optical birefringent patterns are preserved. Conductive, anisotropic hybrid films (5 μm) were obtained by blade coating with concentrated 10wt.% CNT/CNC dispersions. Different percolation models according to the observation of its final microstructure pattern have been applied and fit to the hybrid film conductivities based on the hybrid film structure. SWNT/CNC hybrid film possess a percolation threshold of 0.05%, which is one order of magnitude lower than that of MWNT/CNC hybrid film. Also, comparing MWNT/CNC hybrid film with an MWNT aggregation size of 3 μm and cellular structure of 30 μm with well-dispersed SWNT/CNC hybrid film would be more suitable for conductive device fabrication requiring nano- or micro-scale resolution and uniformity.

## Supplementary Materials

The following are available online at https://www.mdpi.com/1420-3049/24/14/2662/s1, Figures S1–S6.
