# Supplementary material for: Carbon Nanotube and Cellulose Nanocrystal Hybrid Films
Source: Molecules. 2019 Jul 23;24(14):2662. doi: 10.3390/molecules24142662 (PMC6681000; doi:10.3390/molecules24142662)
Supplement: Supplementary file 1 [file molecules-24-02662-s001.pdf]

Carbon Nanotube and Cellulose Nanocrystal Hybrid Films

Mingzhe Jiang, Robert Seney, Paul Charles Bayliss, Christopher L. Kitchens *

Department of Chemical and Biomolecular Engineering, Clemson University
* Correspondence: ckitche@clemson.edu

Supporting Information

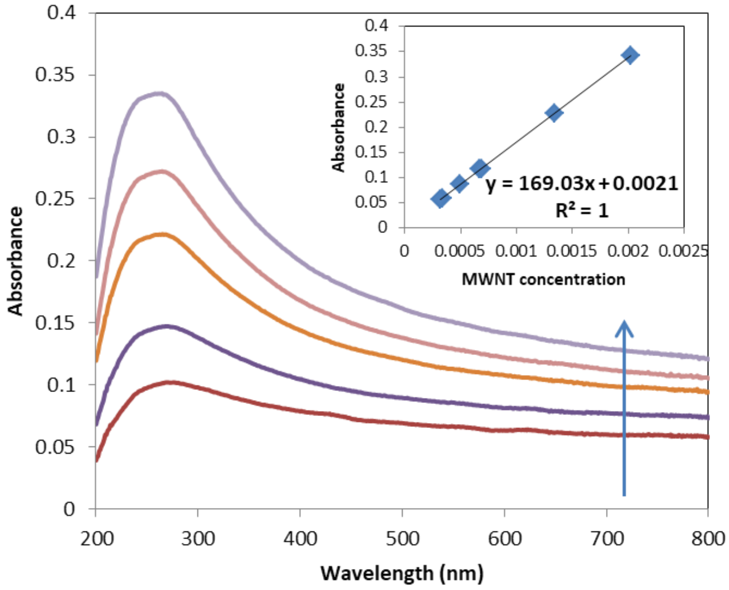

Figure S1. UV–vis spectra of Multi wall carbon nanotubes in CNCs (1%wt) aqueous diserpsion (Beer–Lambert curves inset). Dilute factor: 100. The arrow represents the light wavelength (710 nm) whose absorbance is used for Beer–Lambert curve fitting. The inset is a Beer–Lambert fitting plot of absorbance at 710 nm vs. different concentrations.

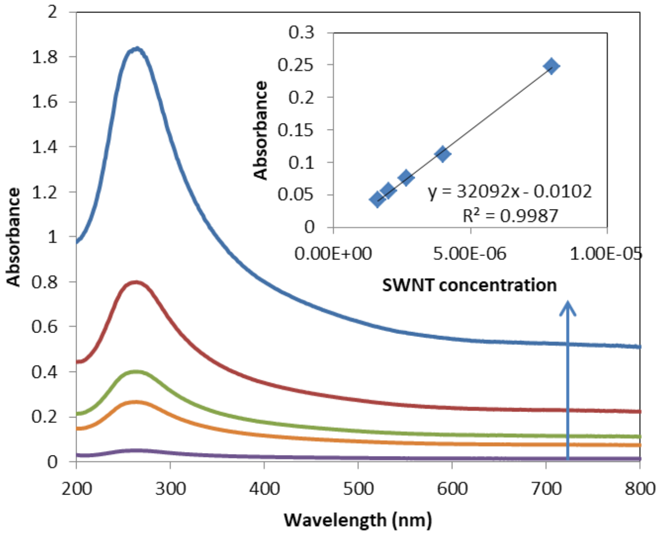

Figure S2. UV–vis spectra of Single wall carbon nanotubes in CNCs (1%wt) aqueous diserpsion (Beer–Lambert curves inset). Dilute factor: 100. The arrow represents the light wavelength (710 nm) whose absorbance is used for Beer–Lambert curve fitting. The inset is a Beer–Lambert fitting plot of absorbance at 710 nm vs. different concentrations.

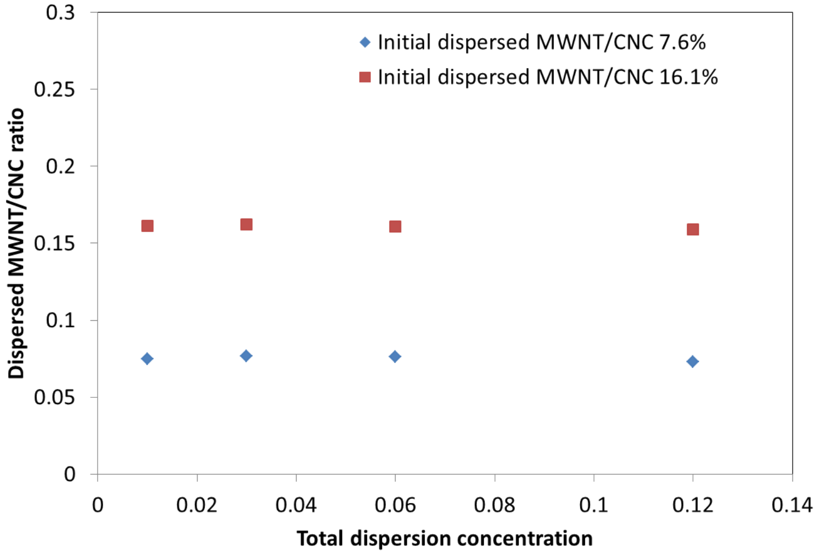

Figure S3. Total dispersion concentration vs. effective dispersed MWNT/CNC ratio

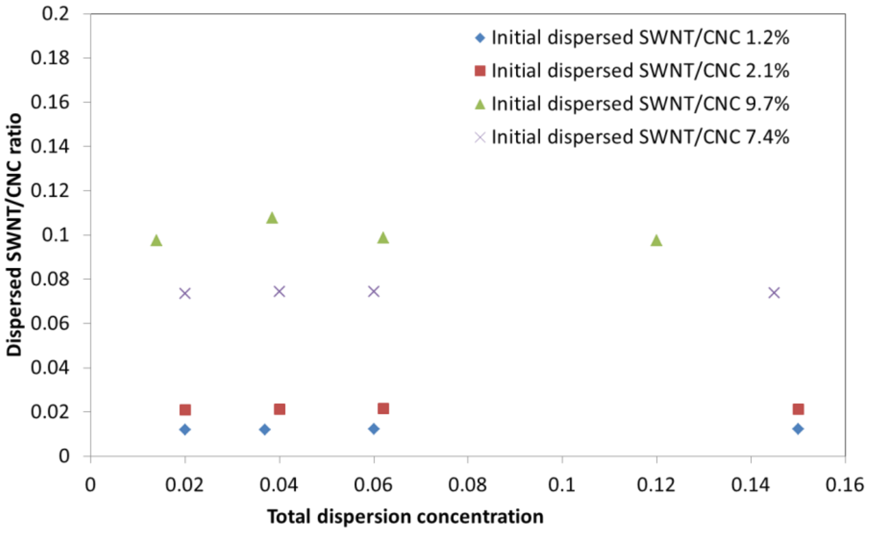

Figure S4. Total dispersion concentration vs. effective dispersed SWNT/CNC ratio

The resulting aqueous MWNT/CNC and SWNT/CNC mixture dispersion was increased by evaporating the water to reach the desired concentration. In order to examine if there is any irreversible aggregation formation as the mixture concentration increases, the concentrated dispersion has been diluted again and put into UV–vis spectrascopy to determine the dispersed CNT concentration. The Figure S3 and 4 shows that the dispersion concentration of MWNT and SWNT in all concentrated mixture remains almost unchanged comparing the initial made mixture dispersion. It proves the total concentration of the mixture play no effects on irreversible aggregation formation and the mixture of both MWNT/CNC and SWNT/CNC maintain as stable dispersion as the total concentration reaches into 12 wt%.

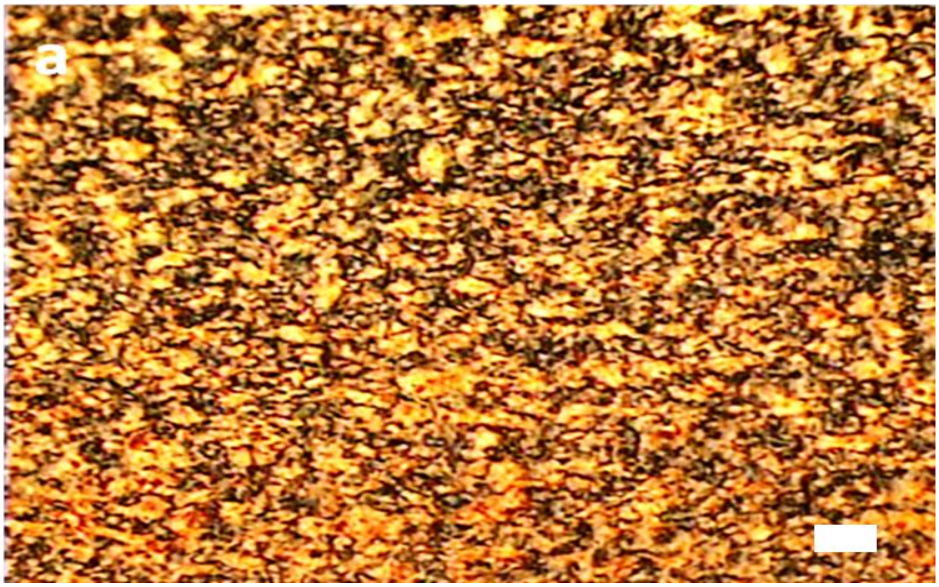

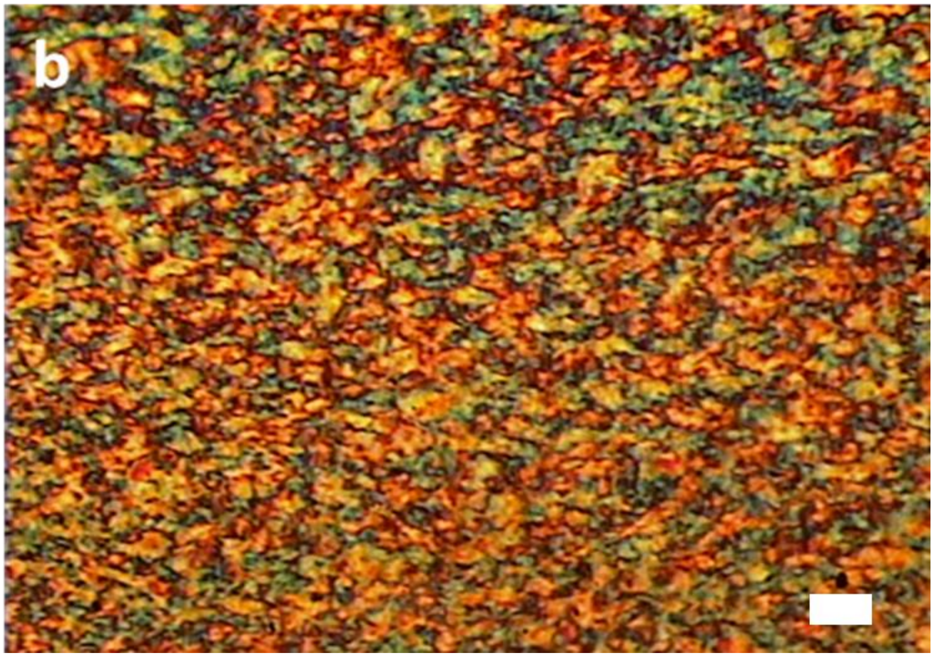

Figure S5. Optical microscope images of 8%wt. CNC with 0.16%wt. SWNT sedated dried film showing schlieren birefringence texture. The images include cross polarized light (a) and cross polarized light with full-wave plate (b). Scale bar: 20 μm.

A retardation filter is inserted between the sample and the analyzer to study the specific orientation of the axis of the liquid crystal (LC). A first order (full wave) plate introduces a relative retardation of one wavelength, allowing visibility of the colors blue and yellow for LC material oriented at 45° and -45°, and magenta for the 0, 90° and disoriented material. In figure C.5, each schlieren brush showed almost-pure color of either blue or yellow in the polarized microscope image with first order retardation plate. This is evidence of nematic orientation at local scale.

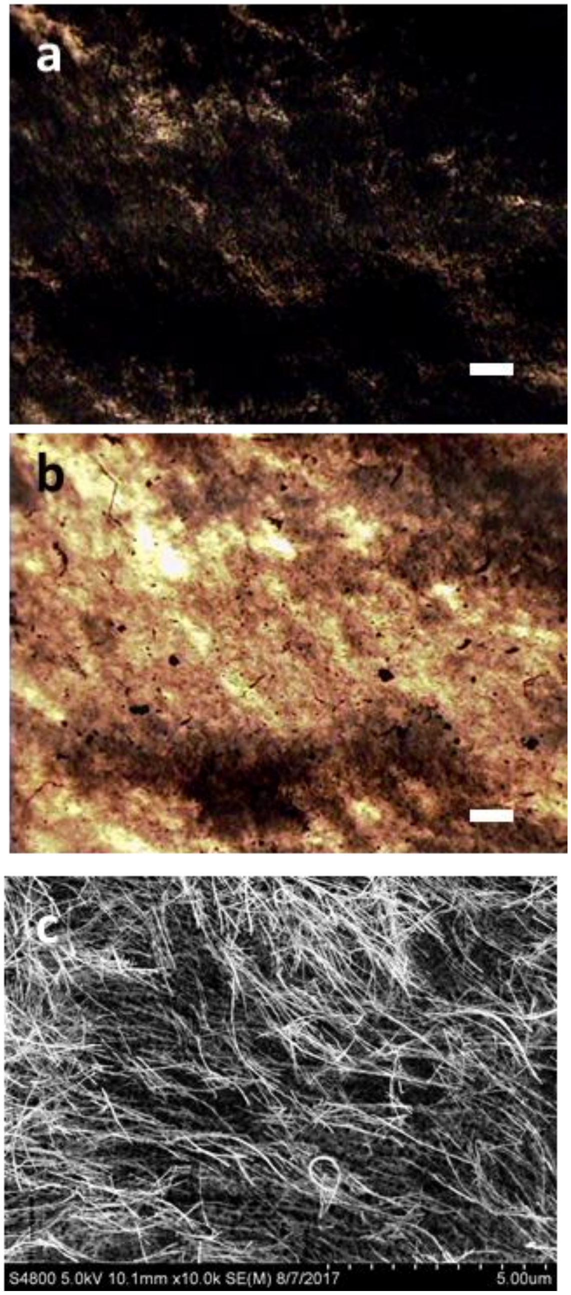

Figure S6. Microscope images of CNC&SWNT (10% SWNT and 90% CNC) hybrid films. a is the cross polarized microscope image, b is the normal light microscope image, c is Scanning Electron Microscope images of CNC&MWNT composite films without platinum coated.

The macroscopic network of nanotubes aggregates of CNC&SWNT hybrid film(10% SWNT and 90% CNC) is seen from the optical image of Figure S7b. Also, no birefringent color is observed from the polarized microscope image. It, together with the misoriented SWNTs in SEM image of Figure S.6c proves that there is agglomerated SWNT fractal network forming in high SWNT loading hybrid film. The SEM image clearly shows that the conductive percolative paths have been increased by the multi-site contact of SWNTs in the agglomerates.
